# How It Seems to Be Struck by Lightning

**Published:** 1892-01

**Authors:** 


### HOW IT SEEMS TO BE STRUCK BY LIGHTNING.

The following interesting account comes from Miss Mary A. C. Avery, of Ledyard, Conn.

I was struck by lightning about 11 o'clock Sunday morning, October 19, 1890. We live a mile and a half from church, and so always ride. That morning it threatened rain, but we went as usual. When near the "green" it thundered and began to rain; so we drove quickly under the first shed to wait until the shower passed, as we knew we were in good season. While sitting in the carriage—mother and I on the same seat, and father between us on a small extra seat—the crash came, but I neither heard it nor saw the lightning. The church, row of sheds, and store were all included in the path of the current. Our horse began floundering, and father sprang to free him from the carriage, lest we be hurt by his kicking. Mother scrambled out as best she could. She says she remembers noticing that I said nothing during the confusion, but she attributed it to my courage with a horse. Presently father, looking up, saw me toppled over in my place, and, calling out that I was hurt, they secured help to carry me to a house, where they dashed on water, and after a time gave me brandy and water. My body was limp and no pulse could be felt. At the end of half an hour, I revived enough to say, "Where am I? Take me home," though I have no remembrance of that; and during all that blank period I was in so strong convulsions that two men could not hold me. This goes to prove that the contortions, about which some people raise objections in electrical executions, are not indicative of conscious suffering.

The first realization of illness I had was from the brandy, for we never use such things except in sickness; so I knew I was in a critical state. But the intense pain now felt prevented full remembrance, and so at first I didn't know but that I had been sick for weeks. I was burned from the left eye, over the head, down the neck, and across to the lower edge of my right shoulder-blade. The iron brace in the carriage seat attracted the lightning to one side, else it might have passed down my spine, causing paralysis, if not death. As it was, the doctors said they could not understand how I survived. The wire in my hat and the hat-pin disappeared, and the lining was scorched out; my collar was split in the middle; my clothing, even to jacket and outer shawl, had an irregular hole, two inches by three or four, punched through. The shawl, after a lapse of ten months, still smells of brimstone. The burn at my eye was more of a scorch than burn proper, but the eye was closed and its sight doubtful for a week. The path through my hair—by which I lost enough to make two good-sized switches!—is recovering itself, and the hair is in some places four inches long. As soon as consciousness really returned I had the use of my mind. The burns were deepest across the shoulders—perhaps as large as two hands—but the soreness there was nothing compared with the pain in the nerves of the arms and chest. I was brought home the third day on a litter, and had no set-backs; the burns healed in three weeks, and in four, I was able to get slowly upstairs.

A curious thing now occurred. One half of my face—the side that was *not* hurt—was paralyzed, the line between the sides being as straight as if drawn by rule, and the eyelid needing to be closed by hand for sleep. I am almost well now, though eye and limbs still feel the effects of the slight paralysis. I have no more fear of thunder-showers than other people, nor can I predict them, but I can tell when the wind is north-east by a faint return of pains in arms or chest. There is a scar upon my right shoulder.

The church had a lightning-rod in good order, and it was generally thought to have saved the building from more serious injury. All the glass of the east windows—on the side where the rod came down—was broken, that upstairs being thrown into the church, while that in the vestry was blown outside. A fire started in a rubbish-closet and under the floor, but was put out before it gained much headway.

The row of sheds contains about twenty, and a disused telephone wire ran over them, to connect the store—perhaps six rods away—with a house twelve rods from the other end. Two sheds in the middle, directly back of the church, and one at each end of the row suffered most. This telephone wire carried the lightning to the store, where some gun-powder was perilously near. The ground where the rod was sunk, and that between church and sheds, and a piece of offset in front of the church, were furrowed and torn badly. No scar could be found upon the horse.

